# Clinical Effect of Radiotherapy Combined with Capecitabine after Neoadjuvant Therapy for Rectal Cancer

**DOI:** 10.1155/2021/9972051

**Published:** 2021-06-05

**Authors:** Qibo Zhang, Haibin Teng

**Affiliations:** Department of Nuclear Medicine, Linyi Central Hospital, Linyi 276400, Shandong Province, China

## Abstract

**Objective:**

The purpose of the study was to investigate the clinical effect of radiotherapy combined with capecitabine in rectal cancer patients after neoadjuvant therapy.

**Methods:**

80 rectal cancer patients who underwent neoadjuvant therapy in our hospital from February 2016 to February 2018 were selected as the study subjects and divided into the control group (*n* = 40) and experimental group (*n* = 40) according to the order of admission. Among them, the control group was treated with radiotherapy, while the experimental group was treated with radiotherapy combined with capecitabine. The therapeutic efficacy, CEA levels, the incidence and recurrence rate of adverse reactions, as well as the progression-free survival and survival rate after 2-year treatment were analyzed in the two groups.

**Results:**

The effective rate of treatment in the experimental group of 87.5% (35/40) was significantly higher than 50% (20/40) in the control group, with statistical significance (*X*^2^ = 13.09, *P* < 0.001). After treatment, the CEA levels in the two groups both decreased significantly, and the CEA level in the experimental group of 3.75 ± 1.76 ng/ml was significantly lower than 7.35 ± 2.11 ng/ml in the control group, with statistical significance (*T* = 8.29, *P* < 0.001). The incidence and the recurrence rate of adverse reactions of 5% (2/40) and 10% (4/40), respectively, in the experimental group were significantly lower than those of 40% (16/40) and 30% (12/40) in the control group, with statistical significance (*X*^2^ = 14.05, 5.00, *P* < 0.001, 0.05). After the 2-year follow-up, it was found that the progression-free survival of 21.53 ± 6.23 months in the experimental group was significantly longer than that of 18.18 ± 5.41 months in the control group, with statistical significance (*T* = 2.57, *P* < 0.05), and the 2-year survival rate of 97.5% (39/40) in the experimental group was significantly higher than 80% (32/40) in the control group, with statistical significance (*T* = 6.13, *P* < 0.05).

**Conclusion:**

Radiotherapy combined with capecitabine in rectal cancer patients after neoadjuvant therapy can improve the therapeutic efficacy with fewer adverse reactions and longer patients' survival, which is worthy of popularization and application after neoadjuvant therapy for rectal cancer.

## 1. Introduction

Rectal cancer is a clinically common malignant tumor disease [1]. The location of rectal cancer is at the intersection of rectosigmoid colon and dentate line, which makes the anatomy more complex, with more difficulty in surgery. The majority of rectal cancer patients are middle-aged, and they are gradually progressing towards a younger age [2–4]. At present, the pathogenic factors of rectal cancer are still unclear, but mainly might be related to diet, society, and heredity. Because rectal cancer patients have no obvious symptoms in the early stage, with the continuous progression of disease, patients will have diarrhea, bloody stools, and other symptoms, which seriously threatens patients' health [5, 6]. In recent years, neoadjuvant therapy has played an active role in the clinical treatment of rectal cancer, but its clinical effect for rectal cancer patients is not ideal. With the continuous development of medical technology, radiotherapy is still an essential part of neoadjuvant therapy for rectal cancer, which can effectively inhibit tumor growth and improve patients' life quality. Capecitabine is an antimetabolic fluorouracil deoxynucleotide-based allophanate ester that is converted into 5-FU in vivo [7], which can inhibit protein synthesis and interfere with RNA and cell division. Nowadays, it has been widely used in the clinical treatment of breast cancer, gastric cancer, and rectal cancer [8]. This study intended to investigate the clinical effect of radiotherapy combined with capecitabine in rectal cancer patients after neoadjuvant therapy and provide some references for rectal cancer treatment.

## 2. Materials and Methods

### 2.1. General Information

The patients with rectal cancer who underwent neoadjuvant therapy in our hospital from February 2016 to February 2018 were selected as the study subjects and divided into the control group and experimental group according to the order of admission. There were no significant differences between the two groups in general clinical data such as age and pathological classification, with comparability (*P* > 0.05), as given in Table 1.

### 2.2. Inclusion/Exclusion Criteria

#### 2.2.1. Inclusion Criteria

1. Patients who met the WHO diagnostic criteria for rectal cancer were clinically diagnosed as rectal cancer in our hospital and received neoadjuvant therapy
2. Patients were or elder than 18 years old
3. This study has been approved by the hospital ethics committee (no. 20160145)
4. Patients and their family were informed of the whole process of treatment and signed the informed consent

#### 2.2.2. Exclusion Criteria

1. Patients had major organ diseases such as kidney, liver, and heart diseases
2. Patients had mental, cognitive, and behavioral disorders
3. Patients had the history of drug allergy
4. Patients had inflammatory bowel disease and hereditary colorectal cancer

### 2.3. Methods

All patients were treated with radiation therapy after neoadjuvant therapy. The radiation instrument Varian Clinac CX 10MV medical electronic linear accelerator was adopted in this study. After simulated positioning, CT enhanced scanning was performed, with the slice thickness of 5 mm. In the emphasized planning system, coplanar multifield conformal irradiation was performed, with the total dose of 60 Gy∼66 Gy (2 Gy/d, 1 time/d, 5 times/week). On the basis of that, the experimental group was treated with Xeloda capecitabine tablets (State Food and Drug Administration approval number: H20073024; manufacturer: Shanghai Roche Pharmaceutical Co., Ltd.), with a total daily dose of 2500 mg (1250 mg/time, once 30 minutes after breakfast and dinner by oral administration). After 14 days of continuous administration, the drug was discontinued for 7 days and 2 courses of treatment were carried out in succession with each course lasting 21 days.

### 2.4. Evaluation Indexes

#### 2.4.1. Therapeutic Efficacy

The therapeutic efficacy was analyzed according to the response evaluation criteria in solid tumors which were divided into complete remission, partial remission, stable disease, and disease progression. Complete remission refers to the disappearance of all tumor lesions for 28 days. Partial remission refers to the reduction of tumor lesion long diameter by more than 30% for 28 days. Stable disease refers to the increase of tumor lesion long diameter by less than or equal to 20% or the reduction by less than or equal to 30%. Disease progression refers to the increase of tumor lesion long diameter more than 20%. The effective rate of treatment = (complete remission + partial remission)/total number of cases × 100%.

#### 2.4.2. CEA Level

The CEA (serum carcinoembryonic antigen) levels before and after treatment were analyzed and compared between the two groups.

#### 2.4.3. Incidence of Adverse Reactions

Adverse reactions such as nausea, vomiting, hand-foot syndrome, diarrhea, myelosuppression, and mucocutaneous impairments were analyzed and compared between the two groups.

#### 2.4.4. Recurrence Rate

The recurrence rate in the two groups of patients was analyzed and compared.

#### 2.4.5. Progression-Free Survival and Survival Rate

Two-year follow-up was carried out for the two groups of patients, and the progression-free survival and 2-year survival rate in the two groups were analyzed.

### 2.5. Statistical Treatment

The data software SPSS18.0 was adopted in this study to process and analyze the research data. Measurement data were expressed by $\left(\bar{x}\pm s\right)$ and tested by the *t*-test. Enumeration data were expressed as (*n* (%)) and tested by the *X*^2^ test. The differences had statistical significance when *P* < 0.05.

## 3. Results

### 3.1. Analysis of Therapeutic Efficacy in the Two Groups of Patients

The effective rate of treatment in the experimental group was significantly higher than that in the control group, with statistical significance (*X*^2^ = 13.09, *P* < 0.001), as given in Table 2.

### 3.2. Analysis of CEA Levels in the Two Groups of Patients

**Figure 1:**
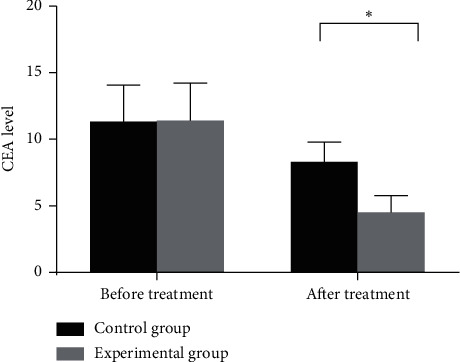
CEA levels in two groups. Note: the abscissa from left to right indicates before treatment and after treatment, while the ordinate indicates the CEA level (unit: ng/ml). The CEA level in the experimental group is significantly lower than that in the control group after treatment. ^*∗*^Statistical significance (*T* = 8.29, *P* < 0.001).

Before treatment, there were no significant differences in CEA levels between the experimental group of 9.53 ± 3.96 ng/ml and the control group of 9.49 ± 3.86 ng/ml (*T* = 0.05, *P*=0.96), with no significant differences. After treatment, CEA levels in the two groups both decreased significantly, and the CEA level of 3.75 ± 1.76 ng/ml in the experimental group was significantly lower than 7.35 ± 2.11 ng/ml in the control group, with statistical significance (*T* = 8.29, *P* < 0.001), as shown in Figure 1.

### 3.3. Analysis of the Incidence of Adverse Reactions in the Two Groups of Patients

The incidence of adverse reactions in the experimental group was significantly lower than that in the control group, with statistical significance (*X*^2^ = 14.05, *P* < 0.001), as given in Table 3.

### 3.4. Analysis of Recurrence Rate in the Two Groups of Patients

**Figure 2:**
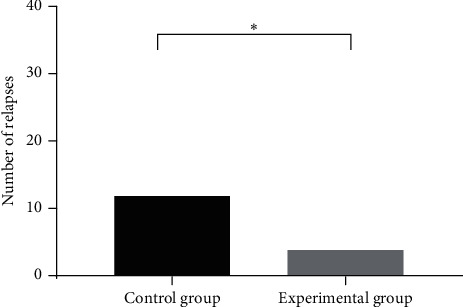
Groups of patients with recurrence rate. Note: the abscissa indicates the control group and experimental group, while the ordinate indicates number of relapses (unit: case). The number of relapses in the experimental group is significantly less than that in the control group. ^*∗*^Statistical significance (*X*^2^ = 5.00, *P* < 0.05).

The recurrence rate of 10% (4/40) in the experimental group was significantly lower than 30% (12/40) in the control group, with statistical significance (*X*^2^ = 5.00, *P* < 0.05), as shown in Figure 2.

### 3.5. Analysis of Progression-Free Survival and Survival Rate after 2-Year Treatment in the Two Groups of Patients

**Figure 3:**
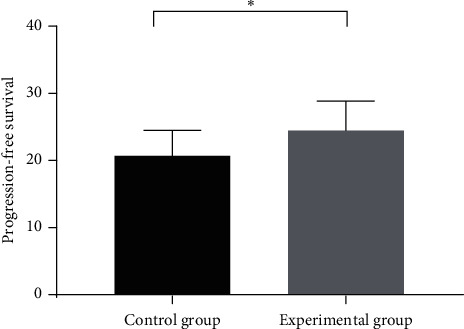
Progression-free survival of the two groups. Note: the abscissa from left to right indicates the control group and experimental group, while the ordinate indicates progression-free survival (unit: month). The progression free survival in the experimental group was significantly longer than that in the control group. ^*∗*^Statistical significance (*T* = 2.57, *P* < 0.01).

**Figure 4:**
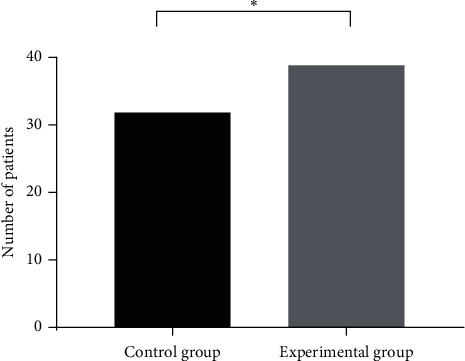
2-year survival rate of the two groups. Note: the abscissa from left to right indicates the control group and experimental group, while the ordinate indicates number of patients (unit: case). The 2-year survival rate in the experimental group was significantly higher than that in the control group. ^*∗*^Statistical significance (*T* = 6.13, *P* < 0.05).

After two-year follow-up, it was found that the progression-free survival of 21.53 ± 6.23 months in the experimental group was significantly longer than 18.18 ± 5.41 months in the control group, with statistical significance (*T* = 2.57, *P* < 0.05). The 2-year survival rate of 97.5% (39/40) in the experimental group was significantly higher than 80% (32/40) in the control group, with statistical significance (*T* = 6.13, *P* < 0.05), as shown in Figures 3 and 4.

## 4. Discussion

Rectal cancer is a clinically common malignant tumor disease, which commonly affects rectum [9–11]. Because rectal cancer patients have no specific clinical symptoms in the early stage, with the progression of disease, patients will suffer from different degrees of constipation and diarrhea. Nowadays, rectal cancer diseases are mostly detected by proctoscope and digital rectal examination, and rectal cancer masses are characterized by rapid growth, uneven surface, and hard texture [12–14]. Surgical resection is currently one of the most commonly used methods in the clinical treatment of rectal cancer, but because most patients are diagnosed in the middle and late stage, the clinical efficacy of surgical resection is not ideal, with high postoperative recurrence rates. In recent years, with the continuous development of medical level, neoadjuvant therapy has been widely applied in clinical treatment. The study of Bushati et al. [15] has found that neoadjuvant therapy for patients with advanced rectal cancer can improve their life quality. At present, most scholars have pointed out that attention should be paid to the following two aspects in the adjuvant treatment of rectal cancer patients. One is that in the process of neoadjuvant treatment, individual differences of rectal cancer patients should be paid much attention to, and disease stages, physical fitness, and treatment compliance of different patients should also be taken into consideration. Another is that radiotherapy or chemotherapy alone is not as effective as chemoradiotherapy because rectal cancer patients have distant metastases during the treatment. Therefore, chemoradiotherapy should be adopted for patients [16–18]. Although neoadjuvant therapy can improve patients' life quality, the recurrence rate after treatment is still very high and the survival rate is not ideal.

Capecitabine is a new 5-FU prodrug. After oral administration, patients convert fluoropyrimidine compounds into 5-FU under the action of thymidine phosphorylase (TP) in vivo. Due to the higher concentration of TP in tumor cells and the lower concentration of TP in normal cells, the activity of TP in tumor cells can be enhanced by radiotherapy, and the effect of 5-FU can be further improved. Therefore, radiotherapy combined with capecitabine in rectal cancer patients after neoadjuvant therapy has great advantages [19, 20]. In recent years, many scholars have proposed that radiotherapy combined with capecitabine in rectal cancer patients after neoadjuvant therapy can reduce patients' adverse reactions and has a positive effect on improving patients' survival rate [21, 22]. In this study, in order to investigate the clinical effect of radiotherapy combined with capecitabine in rectal cancer patients after neoadjuvant therapy, the patients in the control group were treated with radiotherapy, while the patients in the experimental group were treated with radiotherapy combined with capecitabine. The results showed that the therapeutic efficacy and CEA level in the experimental group were significantly better than those in the control group, with statistical significance (*P* < 0.05), indicating that the radiotherapy combined with capecitabine can reduce the infiltration degree of tumor cells in patients.

Neoadjuvant therapy might increase adverse reactions, decrease therapeutic tolerance, and increase the recurrence rate in rectal cancer patients. This study found that the recurrence rate and the incidence of adverse reactions in the experimental group were significantly lower than those in the control group, with statistical significance (*P* < 0.05), which was similar to the conclusion of Yu et al. [23] and others. According to their study, radiotherapy combined with capecitabine under the basis of neoadjuvant therapy increased the safety of treatment and reduced the adverse reactions as well as recurrence rate of patients, which fully demonstrated that radiotherapy combined with capecitabine can reduce adverse reactions.

According to Velenik et al. [24], radiotherapy combined with capecitabine in patients after neoadjuvant treatment of rectal cancer can improve the survival rate. In this study, it was found that the progression-free survival and 2-year survival rate in the experimental group were significantly better than those in the control group, with statistical significance (*P* < 0.05), which indicated that radiotherapy combined with capecitabine can promote tumor regression, thereby improving patients' postoperative survival.

In conclusion, radiotherapy combined with capecitabine in rectal cancer patients after neoadjuvant therapy can improve the clinical efficacy with fewer adverse reactions and longer patients' survival, which is worthy of promotion and application after neoadjuvant therapy for rectal cancer.

## Figures and Tables

**Table 1:** General clinical data of the two groups of patients.

Factors		Control group (*n* = 40)	Experimental group (*n* = 40)	*X* ^2^/*T*	*P*
Gender	Male	22	23	0.05	0.82
	Female	18	17		

Average age	—	55.32 ± 4.75	55.38 ± 4.26	0.06	0.95

Neoplasm staging	Stage II	26	25	0.05	0.82
	Stage III	14	15		

Pathological classification	Mucinous carcinoma	3	4	0.16	0.69
	Adenocarcinoma	37	36		

Differentiation	Poorly differentiated	11	12	0.22	0.90
	Moderately differentiated	15	13		
	Well differentiated	14	15		

**Table 2:** Therapeutic efficacy in the two groups of patients.

Group	Cases	Complete remission	Partial remission	Stable disease	Disease progression	Effective rate of treatment
Control group	40	0	20	15	5	50% (20/40)
Experimental group	40	3	32	5	0	87.5% (35/40)
*X* ^2^	—	—	—	—	—	13.09
*P*	—	—	—	—	—	*P* < 0.01

**Table 3:** Incidence of adverse reactions in the two groups of patients.

Group	Cases	Nausea/vomiting	Hand-foot syndrome	Diarrhea	Myelosuppression	Mucocutaneous impairments	Incidence of adverse reactions
Control group	40	4	2	5	2	3	40% (16/40)
Experimental group	40	1	0	1	0	0	5% (2/40)
*X* ^2^	—	—	—	—	—	—	14.05
*P*	—	—	—	—	—	—	*P* < 0.001

## Data Availability

The data used to support the findings of this study are available from the corresponding author upon request.
